# Bovine Herpesvirus Type 4 (BoHV-4) Vector Delivering Nucleocapsid Protein of Crimean-Congo Hemorrhagic Fever Virus Induces Comparable Protective Immunity against Lethal Challenge in IFNα/β/γR−/− Mice Models

**DOI:** 10.3390/v11030237

**Published:** 2019-03-09

**Authors:** Touraj Aligholipour Farzani, Katalin Földes, Alireza Hanifehnezhad, Burcu Yener Ilce, Seval Bilge Dagalp, Neda Amirzadeh Khiabani, Koray Ergünay, Feray Alkan, Taner Karaoglu, Hurrem Bodur, Aykut Ozkul

**Affiliations:** 1 Virology Department, Faculty of Veterinary Medicine, Ankara University, Ankara 06110, Turkey; touraj.farzani@gmail.com (T.A.F.); fldeskatalin@gmail.com (K.F.); alireza.hanifehnezhad@gmail.com (A.H.); sevalbilge@hotmail.com (S.B.D.); alkanferay@gmail.com (F.A.); tanerkaraoglu@gmail.com (T.K.); 2 Biotechnology Institute, Ankara University, Ankara 06560, Turkey; byener@ankara.edu.tr (B.Y.I.); Neda.Khiabani@hotmail.com (N.A.K.); 3 Virology Unit, Department of Medical Microbiology, Faculty of Medicine, Hacettepe University, Ankara 06100, Turkey; ekoray@hacettepe.edu.tr; 4 Infectious Diseases Clinic, Saglik Bilimleri University, Numune Training and Research Hospital, Ankara 06100, Turkey; hurrembodur@gmail.com

**Keywords:** Crimean-Congo hemorrhagic fever, nucleocapsid, bovine herpesvirus type 4, IFNα/β/γR−/− mice, lethal dose, passive antibody transfer

## Abstract

Crimean-Congo hemorrhagic fever virus (CCHFV) is the causative agent of a tick-borne infection with a significant mortality rate of up to 40% in endemic areas, with evidence of geographical expansion. Due to a lack of effective therapeutics and control measures, the development of a protective CCHFV vaccine remains a crucial public health task. This paper describes, for the first time, a Bovine herpesvirus type 4 (BoHV-4)-based viral vector (BoHV4-∆TK-CCHFV-N) and its immunogenicity in BALB/c and protection potential in IFNα/β/γR−/− mice models in comparison with two routinely used vaccine platforms, namely, Adenovirus type 5 and a DNA vector (pCDNA3.1 myc/His A), expressing the same antigen. All vaccine constructs successfully elicited significantly elevated cytokine levels and specific antibody responses in immunized BALB/c and IFNα/β/γR−/− mice. However, despite highly specific antibody responses in both animal models, the antibodies produced were unable to neutralize the virus in vitro. In the challenge experiment, only the BoHV4-∆TK-CCHFV-N and Ad5-N constructs produced 100% protection against lethal doses of the CCHFV Ank-2 strain in IFNα/β/γR−/− mice. The delivery platforms could not be compared due to similar protection rates in IFNα/β/γR−/− mice. However, during the challenge experiment in the T cell and passive antibody transfer assay, BoHV4-∆TK-CCHFV-N was dominant, with a protection rate of 75% compared to others. In conclusion, vector-based CCHFV N protein expression constitutes an effective approach for vaccine development and BoHV-4 emerged as a strong alternative to previously used viral vectors.

## 1. Introduction

Crimean-Congo hemorrhagic fever virus (CCHFV), a member of the *Orthonairovirus* genus and *Nairoviridae* family, causes a hemorrhagic fever disease with considerable mortality rates in endemic areas such as Turkey [1,2]. The widespread distribution, significant mortality rates and lack of specific treatment or control measures make CCHFV an urgent target for vaccine development [3,4].

The CCHFV nucleocapsid (N) protein is an important structural component with the potential to stimulate humoral and cellular responses following natural or experimental infections. It has, therefore, been targeted for vaccine development using DNA and viral vector platforms in several studies [5,6,7]. During challenge experiments in different modified mice models, such as IFNα/β/γR−/−, immune-suppressed (IS) and STAT-1 knock-out mice, vaccinations based on this protein have demonstrated protective effects in lethal challenges despite the lack of neutralizing antibodies [6,8]. Different expression platforms such as adenovirus and modified vaccinia Ankara (MVA)-based vectors were previously used to deliver N proteins with inconclusive results. A recombinant DNA-based CCHFV-N expression system was previously developed, using modified vaccinia Ankara vectors as the delivery system. However, the immune responses in vaccinated mice were not evaluated in detail and lacked cytokine and antibody response analyses. Although this system seemed to induce antigen-specific immunogenicity in mice, it failed to exert any protective effects upon virus challenge [7].

Considering the disadvantages of routinely used expression platforms, such as pre-existing immunity, the possibility of genome integration, immune responses to the antigens of viral vectors and toxicity related to lytic infections [9], the exploration of new platforms for N protein delivery is a credible approach and likely to accelerate CCHFV vaccine development efforts.

Bovine herpesvirus type 4 (BoHV-4), a member of the *gammaherpesvirinae* subfamily and *rhadinovirus* genus, is prevalent among the cattle population, and can be isolated from healthy animals as well as those with diverse clinical symptoms, although there is no clear evidence linking this virus to any specific pathology [10,11,12]. While the potential of BoHV-4 to infect humans in vivo is obscure, its oncolytic potential based on selective replication strategies in some human cancer cell lines has been demonstrated in vitro [13,14]. This virus has been successfully explored as a novel herpesviral vector in targeting different viral immunodominant antigens, such as the Ebola surface glycoprotein (BoHV-4-syEBOVgD106ΔTK), Peste des Petits Ruminants virus hemagglutinin (H) (BoHV-4-A-PPRV-H-ΔTK) and various glycoproteins (A29L, M1R and B6R) of Monkeypox virus (BoHV-4-A-CMV-A29LgD_106_ΔTK, BoHV-4-A-EF1α-M1RgD_106_ΔTK and BoHV-4-A-EF1α-B6RgD_106_ΔTK) due to some interesting characteristics. These include easy propagation in cell culture systems, a relatively simple genome with a large capacity for inserting foreign genes, availability of an animal model for investigating its pathogenicity (rabbit) and a lack of documented cell transformation in comparison to other *gammaherpesviruses* [15,16,17,18,19,20].

In this study, we generated a recombinant Bovine herpesvirus type 4, expressing the N protein of CCHFV (BoHV4-∆TK-CCHFV-N), and evaluated its immunogenicity in BALB/c and protection potential through a lethal challenge experiment in IFNα/β/γR−/− mice models. Due to the mentioned advantages of this viral vector, we assumed that this novel expression platform could be considered as an alternative in CCHFV vaccination. To test our hypothesis, we further compared the efficacy of BoHV4-∆TK-CCHFV-N with two widely used delivery platforms, including Adenovirus type 5 (Ad5-N) alongside a DNA vector (pCD-N1) expressing the same antigen.

## 2. Methods

### 2.1. Ethics Statement

All animal experiments were performed with the official permission of the Ankara University Ethical Committee for Animal Experiments (17/12/2014; 2014-23-155 and 17/10/2018; 2018-20-130). All animal samplings were conducted according to the national regulations on the operation and procedure of animal experiment ethics committees (Regulation Nr.26220, Date:09.7.2006). During these studies, humane endpoint scores were considered. Multiple observations per day were conducted to confirm the animals’ welfare. Constant access to sterilized water and food was provided. Mice were euthanized by CO_2_ exposure and cervical dislocation. BALB/c and Interferon alpha/beta/gamma receptor-null mice (IFNα/β/γR−/−; AG129; Strain: 129S7/SvEvBrd) were provided by B&K Universal Ltd. (Marshall Bioresouces, Hull, East Yorkshire, UK).

### 2.2. Viruses and Cells

A bovine herpesvirus type 4 (BoHV-4)-carrying bacterial artificial chromosome vector (BoHV4-BAC) was developed from the Movar33/63 European strain by the insertion of a F plasmid (pBeloBAC11; NEB, MA, USA) containing a bacterial and mammalian selection cassette (loxp-BAC-EGFP-loxp) between the open reading frame 2 (ORF2) and ORF3 genes (GenBank Accession Number: NC002665), which are not essential for virus replication, by in vitro homologous recombination in Madin–Darby bovine kidney (MDBK) cells and the infectious BAC (iBAC or BoHV4-BAC). The CCHFV Ank-2 strain (GenBank Accession Number: MK309333; third passage in SW-13 cells) was used as the challenge virus. CCHFV and BoHV4-BAC were cultivated in Scott and White No. 13 (SW-13) and MDBK cells, respectively. SW-13 and MDBK cells were cultured in Eagle’s Minimum Essential Medium (EMEM; Sigma, St. Louis, MO, USA) and Leibovitz’s L-15 (Thermo Fisher Scientific, Waltham, MA, USA) media, supplemented with 10% fetal bovine serum (FBS; Biological Industries, Kibbutz Beit-Haemek, Israel), 2 mM L-glutamine (Biological Industries, Kibbutz Beit-Haemek, Israel), 100 U penicillin, and 0.1 mg/mL streptomycin (Thermo Fisher Scientific). Baby hamster kidney (BHK21-C13) and MDBK stably expressing Cre recombinase enzyme (MDBK-cre) cells were used in the transfection assays and loxp-BAC-EGFP-loxp elimination from the BoHV4-BAC construct, respectively. Cultivation of these cell lines was the same as for MDBK cells. In addition, we performed preliminary experiments using human embryonic kidney (HEK293 and HEK293A) cells for the generation and cultivation of the recombinant adenovirus type 5 expressing CCHFV N protein. These cells were cultured and maintained at 37 °C/5% CO_2_ in Minimum Essential Medium alpha (Thermo Fisher Scientific). The final N-expressing constructs, including BoHV4-∆TK-CCHFV-N and Ad5-N, were propagated and titered in MDBK and HEK293A cells, respectively, and stored at −80 °C. All viruses and cells were obtained from the departmental collection. All biological assays, including virus cultivation and animal experiments with infectious virus particles were performed in the biosafety level 3 plus (BSL3+) and animal biosafety level 3 plus (ABSL3+) facilities of the Virology Department, Veterinary Faculty of Ankara University, Turkey.

### 2.3. Recombinant BoHV4-∆TK-CCHFV-N

CCHFV (Ank-2 strain) genomic RNA extraction was performed using the QIAamp Viral RNA Mini Kit (QIAGEN, Germantown, MD, USA) according to the manufacturer’s instructions. Superscript IV reverse transcriptase (Thermo Fisher Scientific) was employed for cDNA synthesis. Table 1 shows the primer sets used to amplify the complete N coding gene of CCHFV based on the Turkey-Kelkit06 complete sequence (GenBank Accession Number: GQ337053), TK-CMV-N-TK homologous cassette to the thymidine kinase gene of the BoHV-4 Movar33/63 strain and the S gene PCR product containing the 50 bp homologous arms of the pCDNA3.1 myc/HisA vector. In the first step, the pCD-N1 construct was generated by the insertion of the gel-purified N PCR product between the EcoRI and XhoI sites of the pCDNA3.1 myc/His A plasmid (Invitrogen, Carlsbad, CA, USA). The final construct was verified by sequencing. In the next step, to propagate the TK-CMV-N-TK cassette from pCD-N1, primer sets (TM1 and TM2) containing the 50 bp homologous arms of the thymidine kinase gene (GenBank Accession Number: AF318573) of BoHV4-BAC (iBAC) were used. This cassette was used to perform recombineering in SW102 bacterial cells that previously contained BoHV4-loxp-BAC-CMV-EGFP-loxp (iBAC) following gel purification, according to the standard protocol (Available online: https://redrecombineering.ncifcrf.gov/protocols/) [21]. This bacterial strain is derived from DY380 (DH10B-derived strain) and, therefore, contains a defective λ prophage with the recombination proteins, exo, bet, and gam, under the control of the temperature-sensitive repressor, *cI*857. To perform recombineering, the gel-purified TK-CMV-N-TK cassette (100 ng) was electroporated into heat-induced SW102 (42 °C for 15 min) containing iBAC in a 0.1-cm cuvette using a micropulser electroporator (BioRad, Hercules, CA, USA), and was subsequently plated on Luria–Bertani (LB) agar containing 12.5 µg/µL of chloramphenicol. Following the extraction of the verified BoHV4-BAC-∆TK-CCHFV-N DNA from SW102 by the alkaline lysis method, the MDBK cells were transfected using Lipofectamine 3000 reagent (Thermo Fisher Scientific) to generate infectious BoHV4-BAC-∆TK-CCHFV-N viruses. To eliminate loxp-BAC-EGFP-loxp from the final construct, recombinant BoHV4-BAC-∆TK-CCHFV-N was propagated in MDBK-cre cells to create BoHV4-∆TK-CCHFV-N viruses.

### 2.4. Recombinant Ad5-N

The AdMax™HI-IQ Kit J (MICROBIX Biosystems, Ontario, Canada) was used to generate Ad5-N. Basically, the S gene was cloned into the Ad5 shuttle vector (pDC516), which was flanked at 5′ by the Cytomegalovirus promoter (CMV) and at 3′ by an SV40 polyadenylation signal sequence, and contained E1 homologous arms to the pBHGfrt∆E1 plasmid. The seamless ligation cloning extract (SLiCE) method was used, which includes PCR amplification by primers targeting the S gene with the 50 bp homologous arms at the 5′ and 3′ sides of the EcoRI of the pDC516 vector, according to the standard protocol [20]. Table 1 shows the employed primer set 1. The SLiCE extract was prepared from PPY bacteria (DH10B-derived *E. coli* strain:F^−^ *end*A1 *rec*A1 *gal*E15 *gal*K16 *nup*G *rps*LΔ*lac*X74*Φ80lac*ZΔ M15*ara*D139Δ(*ara,leu*)7697 *mcr*AΔ(*mrr-hsd*RMS-*mcr*BC) *cyn*X: [*ara*CpBAD*-redα* EM7-*redβ*Tn5-*gam*] *λ^−^*) as described elsewhere [22]. The final construct (pDC516-N) was confirmed by colony PCR, restriction enzyme analysis and sequencing of the target gene. To rescue recombinant viruses, homologous recombination was achieved with the co-transfection of the pDC516-N (5 µg) and pBHGfrt∆E1 (3 µg) plasmids, using Lipofectamine 3000 reagent in a T25 cell culture flask containing HEK293 cells at a confluency of 90%. Twenty-four hours post-transfection, the cells were harvested by trypsin and transferred to a T75 culture vessel. The viruses were collected after 12–14 days. The cytopathic effects (CPEs), including rounded and detached cells, were then visualized in 90% of the monolayer. After 3 rounds of virus passage in HEK293 cells, a sufficient titer of the recombinant viruses was obtained. The recombinant viruses were titered in HEK293A cells by Adeno-X™ Rapid Titer Kit (Clontech, CA, USA). In addition, Ad5-wt virus was produced by the transfection of the pFG140 vector in HEK293 cells. Following titer determination, the virus stocks were stored at −80 °C.

### 2.5. In Vitro Detection of N Protein Expression

To confirm the in vitro expression of N proteins from the pCD-N1 and pDC516-N constructs, an indirect immunofluorescence assay (IIFA) was performed in BHK21-C13 cells, as previously described [23]. Briefly, BHK21-C13 cells were cultivated in 24-well plates for 24 h before pCD-N1 transfection by Lipofectamine3000. The cells were fixed by 3.7% formaldehyde 48 h post-transfection, blocked with 5% skimmed milk (Cell Signaling, Leiden, The Netherlands) in 1x tris based buffer (TBS) buffer containing 0.2% Tween-20 (1xTBST), and incubated with a 1:250 dilution of primary human anti-CCHFV-N polyclonal antibodies in 1xTBST for 90 min at room temperature (RT). Then, Fluorescein isothiocyanate (FITC) -labeled anti-human IgG secondary antibody (Sigma) at a dilution of 1:750 was added and incubated for 1 h. The assay was evaluated by examining the cells with an Axio Vert A1 Microscope (Ziess, Oberkochen, Germany).

N protein expression in recombinant viruses was evaluated by Western blot assay. Infected MDBK (BoHV4-∆TK-CCHFV-S) and HEK293 (Ad5-N) cells (10 moi) were scraped 96 h post-inoculation and total proteins were extracted by the PRO-PREP™ protein extraction solution (iNtRON Biotechnology, Burlington, Massachusetts, USA), separated in Mini-Protean TGX Stain-Free precast gels (BioRad) in 1xTris/Glycine/SDS buffer and then transferred to a polyvinylidene difluoride (PVDF; BioRad) membrane using the Trans-Blot Turbo Transfer System (BioRad). The membrane was subsequently blocked with 5% skimmed milk in 1xTBST buffer for one hour followed by incubation with primary human polyclonal antibody at a dilution of 1:250 in 1xTBST for 2 hours at RT. Anti-human IgG-HRP secondary antibody (Sigma) at a dilution of 1:750 was added and incubated for an additional hour with gentle shaking. Finally, bands were visualized after the incubation of the membranes in a Clarity Western ECL substrate solution (BioRad) for 10 min in the dark and imaged using the ChemiDoc MP System (BioRad). Anti-Beta Actin antibody (St John’s Laboratory, London, UK) and CCHFV Ank-2-infected SW-13 cells (0.1 moi) at 3 days post-inoculation were used as controls. 

### 2.6. Immunization Schedule of BALB/c Mice

A total of twenty-four female BALB/c (8–10 weeks old) mice were used in the study (Table 2). The animals were randomly divided into six groups of four individuals, comprising BoHV4-∆TK-CCHFV-N, Ad5-N, Movar33/63, Ad5-wt, pCD-N1 and pCDNA3.1 myc/His A and a negative control (normal saline). At day 0, the study groups received 100TCID_50_/0.3 mL doses of the relevant viruses through the intra-peritoneal route (i.p.), while the pCD-N1 and pCDNA3.1 myc/His A groups were injected with 50 µg of the constructs through the thigh muscle of the hind limb (i.m.). Booster injections were given after 2 weeks of the same regime. Serum samples were obtained from blood collected through the tail vein on days 0, 14 and 28, and stored at −80 °C until further analysis. In addition, splenocytes from individual mice were collected on day 28 for cytokine analysis.

### 2.7. Immunization and Challenge Experiment of IFNα/β/γR−/− Mice

The CCHFV Ank-2 strain, which was previously demonstrated to be lethal for IFNα/β/γR−/− mice, was used in the intra-peritoneal challenge experiment [23]. The immunization schedule (vaccine constructs and their respective backbones) of IFNα/β/γR−/− mice (8–12-week-old female) was similar to BALB/c mice. All animals (4 animals/group) received 100TCID_50_/300 µL of recombinant/wild type viruses and 50 µg/100 µL of DNA constructs (pCD-N1 and pCDNA3.1) on days 0 and 14. Serum samples were collected on days 0, 14, 28, prior to the challenge and stored at −80 °C to perform cytokine analysis and ELISA for the detection of N-specific IgG. To perform the challenge experiment, each group received 300 µL of a lethal dose of the virus (100LD_50_ = 1000TCID_50_; third passage in SW-13 cell) on day 28 and the assay continued for 13 days. Daily observations of clinical signs were recorded, including nasal or ocular discharge, appearance change, weight loss, depression and death. The euthanasia criteria for the surviving groups were based on complete recovery from clinical signs and stability of body weight for a minimum of 3 days.

### 2.8. Total Antibody Isotyping Assay from Immunized BALB/c Mice

A total antibody Isotyping ELISA kit (Thermo Fisher Scientific) was used to determine total immunoglobulin isotypes in immunized BALB/c mice serum samples collected on days 0 and 28, as previously described [23].

### 2.9. ELISA from Serum Samples of Immunized IFNα/β/γR−/−

To detect the potential of our N-expressing constructs to induce specific antibody responses in IFNα/β/γR−/− mice (before the challenge experiment), we developed an in-house enzyme linked immunosorbent assay (ELISA). Briefly, infected SW-13 cells with 1 moi of the Ank-2 strain were collected 2 days post-inoculation (dpi) and cell lysates were used as ELISA antigens. A total of 45 µg of the cell lysate was added to each well of a Nunc MaxiSorp flat-bottom (Thermo Fisher Scientific) in a bicarbonate buffer (pH 8) and incubated overnight at 4 °C. The next day, 1/1000 diluted serum samples (on day 28) from immunized IFNα/β/γR−/− mice were added to each well and incubated for 2 h at room temperature (RT). Subsequently, anti-mouse IgG-HRP antibody (Abcam, Cambridge, MA, USA) at a dilution of 1/10,000 was added and further incubated at RT for 1 h, followed by adding 3, 3′, 5, 5′-Tetramethylbenzidine (TMB ELISA Peroxidase) substrate. Finally, the reaction was stopped by adding 2N H_2_SO_4_. The results of the unvaccinated mice serum samples were subtracted from the cell lysate as background.

### 2.10. Antibody Passive Transfer and T Cell Adoptive Transfer

Splenocytes and serum samples from immunized BALB/c mice were harvested on day 28 (two weeks after the booster dose). After staining with trypan blue (Thermo Fisher Scientific), 2 × 10^5^ splenocytes combined with 100–300 µL of serum containing 500 µg of IgG antibody (measured by mouse anti-IgG kit, Wuhan Fine Biotech Co., Ltd. Wuhan, China) were injected through the intra-peritoneal route into IFNα/β/γR−/− mice (each group contained four 8- to 12-week-old female mice). Twenty-four hours later, the mice were challenged by a lethal dose (1000TCID_50_/300 µL) of the Ank-2 strain (i.p. route). The challenged animals were observed daily for onset of clinical signs and death. Survival rates and body weight percentages were recorded for a maximum of 15 days.

### 2.11. Virus Neutralization Assay (VNA)

A VNA was employed to elucidate the neutralizing ability of the actively immunized animals. Actively immunized BALB/c and IFNα/β/γR−/− mice serum samples, on days 14 and 28, were heat-inactivated at 56 °C for 30 min in a water bath and then serially diluted (1/2) in Dulbecco’s Modified Eagle’s medium (DMEM) and mixed with an equal volume of 100TCID_50_ of the virus in duplicate. After 1 h of incubation at 37 °C, the serum–virus mixture was inoculated in 1-day-old SW-13 cells at 90% confluency prepared in 24-well plates. The infected cells were further incubated under the same conditions for 4–5 days, with daily observation via an inverted microscope for virus-induced cytopathic effects.

### 2.12. Cytokine Assays

The supernatants collected from the virus-stimulated splenocytes of BALB/c and serum samples of BALB/c (on days 0 and 28) and IFNα/β/γR−/− (on days 0 and 33/34 for control groups/42 for survived groups from N-expressing groups) mice were subjected to cytokine measurements. To measure cytokine responses from virus-stimulated splenocyte supernatants, immunized BALB/c mice were euthanized on day 28 and their spleens were aseptically removed to prepare single cell splenocytes. After cell dissociation, red blood cells were lysed by red blood cell (RBC) lysis buffer (Biological Industries, Kibbutz Beit-Haemek, Israel) and the cells were resuspended in RPMI-1640 media (Sigma) then cultured in 24-well plates (250 × 10^3^ per well). Immediately, 10 moi of Ank-2 strain (100 µL) was added to each well and the supernatants were collected after 48 and 72 h post-infection. The quantitation of cytokines from splenocyte supernatants (BALB/c) and serum samples (BALB/c and IFNα/β/γR−/−) was performed using the LEGENDplex™ Mouse Th1/Th2 Panel 8-plex kit (BioLegend, San Diego, CA, USA) by FacsCanto II Flow Cytometer (BD Bioscience, Franklin Lakes, New Jersey, USA). The results were analyzed by LEGENDplex™ data analysis software based on the manufacturer’s instructions.

## 3. Statistical Analysis

The antibody isotype data and cytokine levels among the groups were evaluated using a two-way (Sidak’s post hoc correction) ANOVA by SPSS (IBM SPSS Statistics for Windows, Version 22.0. Armonk, NY, USA). One-way ANOVA (Tukey’s post hoc correction) was also employed to analyze neutralizing antibody production. Graphs were produced using GraphPad Prism version 7.0 (GraphPad Software, San Diego, CA, USA; www.graphpad.com). Data were considered statistically significant when *p* < 0.05. All molecular biology procedures were simulated using SnapGene Viewer software (www.snapgene.com).

## 4. Results

### 4.1. Recombineering and Homologous Recombination

**Figure 1:**
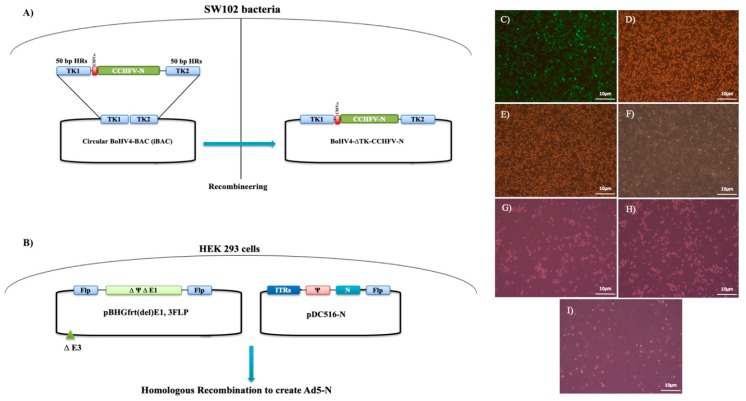
(**A**,**B**) Schematic figure of recombineering in SW102 bacteria to create BoHV-4 viral vector expressing nucleocapsid protein of CCHFV (BoHV4-∆TK-CCHFV-N) (**A**) and a homologous recombination in HEK293 to rescue Ad5-N (**B**). BoHV4-∆TK-CCHFV-N, Movar33/63, Ad5-N and Ad5-wt propagation in cells. (**C**) BoHV4-∆TK-CCHFV-N-infected MDBK cells on day 5 (fluorescence) post-infection (p.i). (**D**) BoHV4-∆TK-CCHFV-N-infected MDBK cells on day 5 (phase contrast) p.i. (**E**) Movar33/63-infected MDBK cells on day 5 p.i. (**F**) Uninfected-MDBK cells on day 5 p.i. (**G**) Ad5-N-infected HEK293 cells on day 5 p.i. (**H**) Ad5-wt-infected HEK293 cells on day 5 p.i. (**I**) Uninfected-HEK293 cells on day 5 (×400). All the viruses were inoculated at 1 moi in the mentioned cells.

The generation of BoHV4-∆TK-CCHFV-N was accomplished in SW102 bacteria containing BoHV4-BAC (Figure 1A). After selection of the right colonies, the constructs were verified via restriction enzyme analysis by comparing them to the Movar33/63 strain. Their stability within the bacteria was determined by 10 bacterial passages. Finally, the extracted DNA of BoHV4-∆TK-CCHFV-N was transfected in MDBK cells to reconstruct the infectious viruses (Figure 1C,D), which were used as vaccine constructs in vivo. The created recombinant BoHV4-∆TK-CCHFV-N showed a similar pattern of CPEs in the MDBK cells compared to its respective virus (Movar33/63; Figure 1E,F). In the homologous recombination experiment, the co-transfection of the pDC516-N and pBHGfrt∆E1 plasmids in HEK293 cells resulted in rescuing of recombinant Ad5-N after 12–14 days post-DNA delivery (Figure 1B). The created Ad5-N reached the sufficient titer after three passages in HEK293A. Finally, the virus was propagated in HEK293 cells (Figure 1G,H) and used in the mice immunization experiments.

### 4.2. In Vitro Verification of N Protein Expression

**Figure 2:**
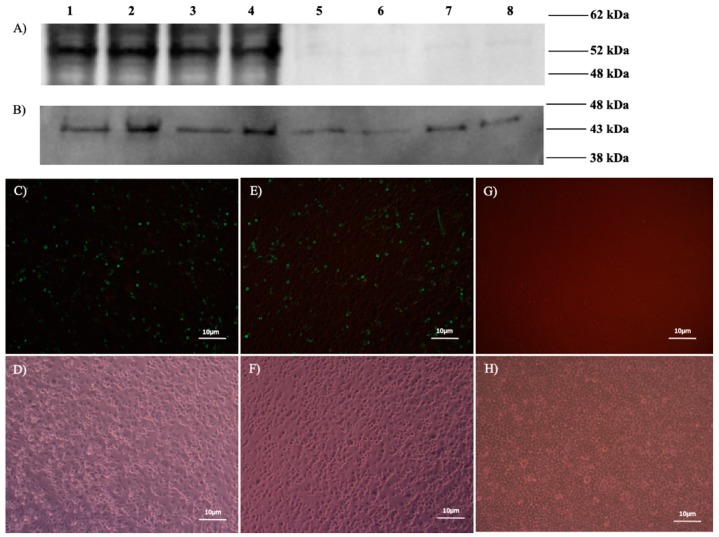
In vitro expression of N protein. (**A**,**B**) Western blot assay (WB). (**A**) Positive bands of 52 kDa (N protein) detected in lane 1 (CCHFV Ank-2-infected SW-13 cells), lane 2 (BoHV4-∆TK-CCHFV-N-infected MDBK cells), lane 3 (Ad5-N-infected HEK293 cells), and lane 4 (pCD-N1-transfected HEK293A cells). We also included control groups of lane 5 (Movar33/63-infected MDBK cells), lane 6 (Ad5-wt-infected HEK293 cells), lane 7 (pCDNA3.1 myc/His A-transfected HEK293 cells) and lane 8 (negative control) in the experiment. (**B**) Beta-actin protein (43 kDa) was detected as the loading control of WB in all mentioned groups. (**C**–**H**) IIFA 48 h post-transfection. (**C**) pCD-N1-transfected BHK21-C13 cells (fluorescence). (**D**) pCD-N1-transfected BHK21-C13 cells (phase contrast). (**E**) pDC516-N-transfected BHK21-C13 cells (fluorescence). (**F**) pDC516-N-transfected BHK21-C13 cells (phase contrast). (**G**) Negative control cells (fluorescence). (**H**) Negative control cells (phase contrast). In IIFA and WB assays for the detection of N proteins, the primary and secondary antibodies are primary human polyclonal (1/250 dilution) and anti-human IgG-HRP secondary antibodies (1/750 dilution), respectively.

The presence of N proteins (~52 kDa) in BoHV4-∆TK-CCHFV-N-infected MDBK, Ad5-N-infected HEK293A and pCD-N1-transfected HEK293A cells was confirmed by immunoblotting (Figure 2A). Ank-2-infected SW-13 cells were used as the positive control for western blot. B-actin protein was detected in all groups, including infected/transfected and negative controls, as a loading control (Figure 2B). N protein expression was verified in pCD-N1- and pDC516-N-transfected BHK21-C13 cells on day 2 post-DNA delivery by indirect immunofluorescence assay (IIFA) (Figure 2C–F). As a negative control, un-transfected-BHK21-C13 cells were included in the assay (Figure 2G,H).

### 4.3. Serological Assays

**Figure 3:**
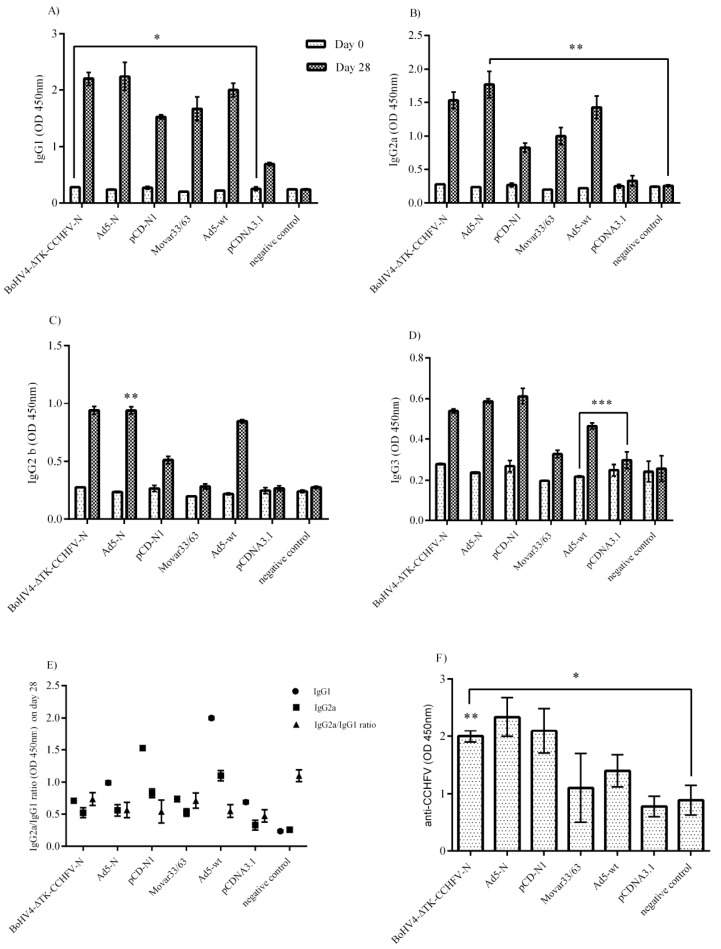
Serological Assays. Total antibody isotype responses and ELISA assays were performed in BALB/c and IFNα/β/γR−/− mice serum samples, respectively. (**A**) IgG1 response: pCD-N1-immunized BALB/c mice showed higher levels of this antibody than other N-expressing constructs. (**B**) IgG2a response: considering the respective backbones, the BoHV4-∆TK-CCHFV-N and pCD-N1 groups had more potential to stimulate IgG2a production. (**C**) IgG2b response: considering the N-expressing groups and related backbone subtraction, the BoHV4-∆TK-CCHFV-N group was dominant. (**D**) IgG3 response: the results were comparable to the IgG1 findings. (**E**) IgG2a/IgG1 ratio (BALB/c mice): all groups except normal saline demonstrated a ratio <1, indicating a shift towards Th2 responses. (**F**) ELISA assay from immunized IFNα/β/γR−/− mice: all constructs expressing N protein could produce N-specific IgG antibodies after a 2-dose injection and before the challenge assay on day 28. The highest amount of specific antibodies was detected in the pCD-N1 group when the results were compared to the respective backbones. In addition, the BoHV4-∆TK-CCHFV-N- and Ad5-N-related findings are also significant. All data are shown as mean ± SD.* *p* < 0.05; ** *p* < 0.01 and *** *p* < 0.001 versus respective backbones.

The BALB/c mice immunized by N-expressing constructs showed elevated levels of IgG1, IgG2a, IgG2b and IgG3 compared to their respective backbones including pCDNA3.1, Movar33/63 and Ad5-wt (Figure 3A–D). As demonstrated, the pCD-N1 construct had more potential for IgG1 and IgG3 production (Figure 3A,C). This construct along with BoHV4-∆TK-CCHFV-N were predominant in inducing IgG2a antibodies (Figure 3B). BoHV4-∆TK-CCHFV-N-immunized mice showed a higher level of IgG2b than the other N-expressing constructs. In addition, the IgG_2a_/IgG_1_ ratio reflected a shift towards Th2 responses in all the immunized BALB/c mice except the normal saline (Figure 3E). In the in-house ELISA assay, all the serum samples from BoHV4-∆TK-CCHFV-N-, Ad5-N- and pCD-N1-immunized IFNα/β/γR−/− mice on day 28 (before the lethal challenge experiment) showed relatively high levels of N-specific IgG antibodies. However, the differences were not significant among the vaccine groups (Figure 3F). Despite the high level of antibody production in the immunized BALB/c and IFNα/β/γR−/− mice, cytopathic effects were observed in SW-13 cells in the neutralization assays, indicating that the produced antibodies were non-neutralizing.

### 4.4. Cytokine Assays

**Figure 4:**
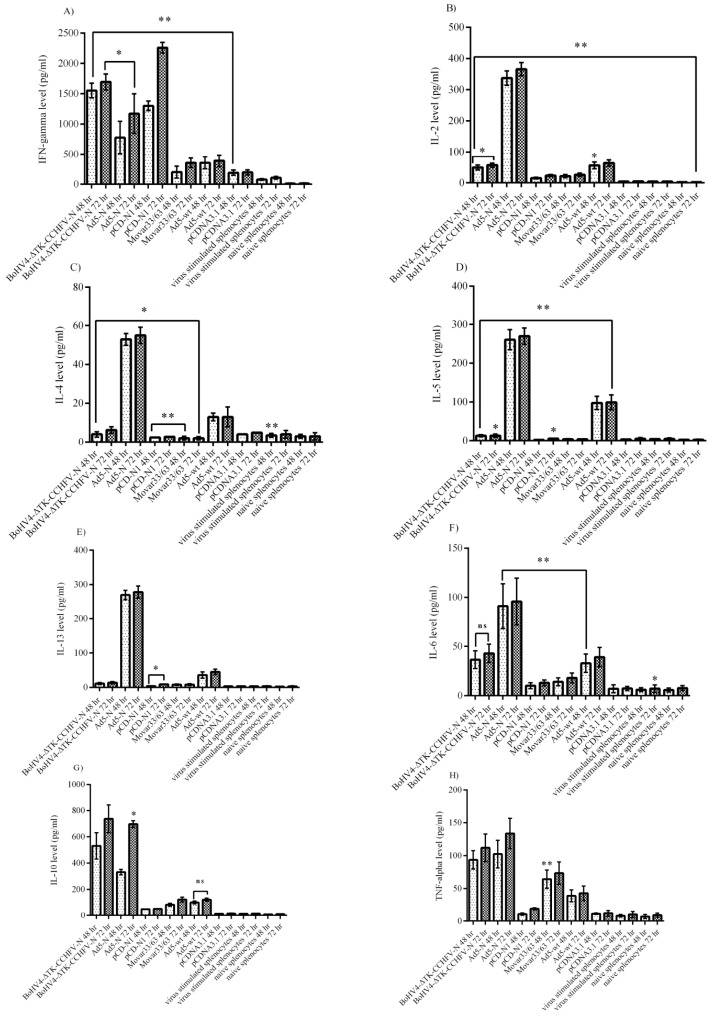
Cytokine responses of Ank-2 virus-stimulated (moi 10) splenocytes from immunized BALB/c mice (on day 28) after 48 and 72 h post-infection. (**A**) IFN-gamma response: as demonstrated here, the pCD-N1 group was higher than other N-expressing groups. (**B**) IL-2 response: Ad5-N was dominant. Other groups could not stimulate adequate production. (**C**) IL-4 response: the results were similar to IL-2. (**D**) IL-5 response: Ad5-N showed more potential to secrete IL-5. (**E**) IL-13 response: the results were similar to IL-2, IL-4 and IL-5. (**F**) IL-6 response: while the Ad5-N group was higher than the BoHV4-∆TK-CCHFV-N group, sufficient levels were also stimulated by this construct. (**G**) IL-10 response: both the BoHV4-∆TK-CCHFV-N and Ad5-N groups induced significant amounts compared to their respective backbone and pCD-N1. (**H**) TNF-alpha response: the results were similar to IL-10. All data are shown as mean ± SD; * *p* < 0.05 and ** *p* < 0.01 versus respective backbones.

The cytokine responses in the virus-stimulated splenocytes of the immunized BALB/c mice were analyzed. The evaluation of the major Th_1_ cytokines in the supernatants of CCHFV-stimulated splenocytes revealed significantly higher levels of interferon gamma in the pCD-N1 and IL-2 in Ad5-N at both 48 and 72 h post-stimulation (Figure 4A,B). BoHV4-∆TK-CCHFV-N showed the potential to elicit the significant amount of IFN-gamma detected in the supernatant. However, this construct could not stimulate IL-2 production in vitro (Figure 4A,B). On the other hand, the Ad5-N group was predominant in IL-4, IL-5 and IL-13 as an indicator of Th2 response in comparison to other N-expressing constructs. In other N-expressing groups, these cytokines (BoHV4-∆TK-CCHFV-N and pCD-N1) were hardly detectable compared to their respective backbones (Figure 4C–E). Ad5-N was also dominant in IL-6 production. In addition, the BoHV4-∆TK-CCHFV-N group’s splenocytes demonstrated relatively significant IL-6 secretion in comparison to the DNA vaccine construct, which could not stimulate the production of this cytokine (Figure 4F). In addition, the Ad5-N and BoHV4-∆TK-CCHFV-N groups showed the potential to stimulate the production of adequate amounts of IL-10 and TNF-alpha in the supernatant samples (Figure 4G,H).

**Figure 5:**
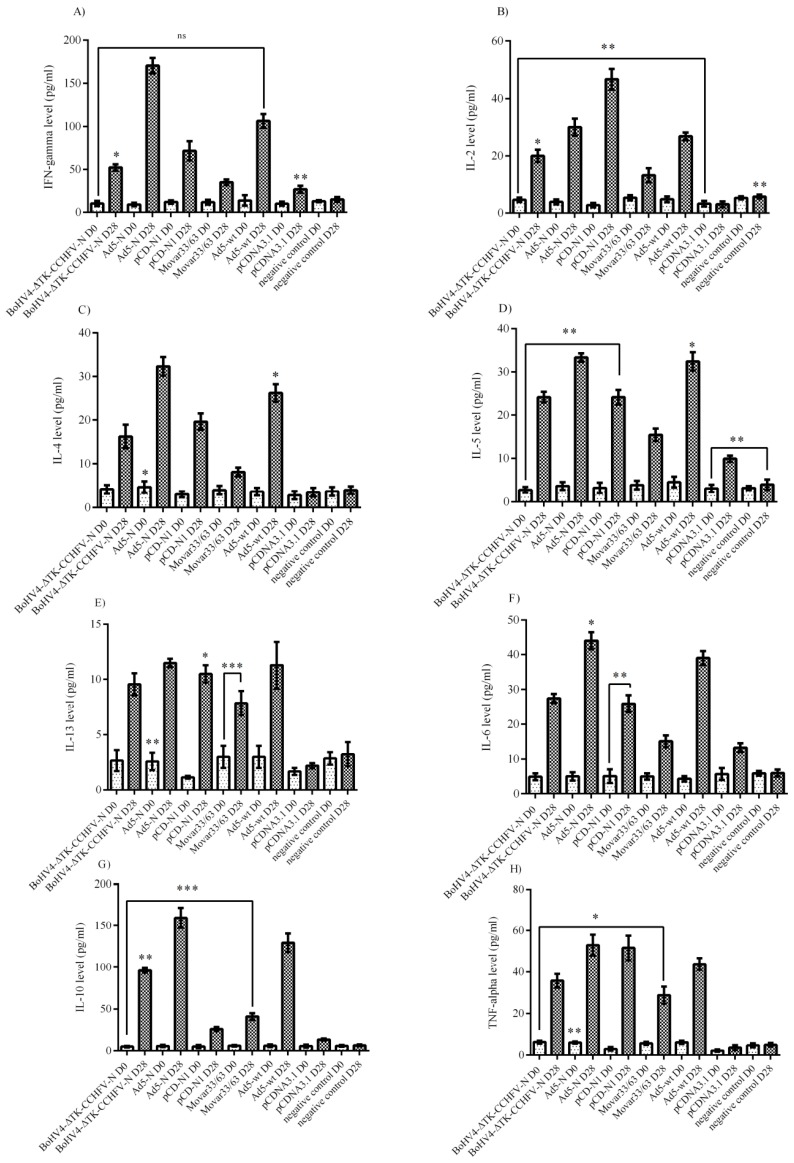
Cytokine responses of immunized BALB/c mice serum samples on days 0 (D0) and 28 (D28). (**A**) IFN-gamma response: the Ad5-N construct was dominant in comparison to other N-expressing ones. (**B**) IL-2 response: considering the respective backbones, the pCD-N1 construct showed increased potential to elicit this cytokine’s production. (**C**) IL-4 response: similar to IL-2 response, the pCD-N1 group showed elevated levels. IL-4 was further significant in the BoHV4-∆TK-CCHFV-N group. (**D**) IL-5 response: identical to IL-4. (**E**) IL-6 response: the BoHV4-∆TK-CCHFV-N and pCD-N1 groups showed higher levels. (**F**) IL-10 response: the BoHV4-∆TK-CCHFV-N group showed higher potential for stimulation. (**G**) IL-13 response: similar to IL-2. (**H**) TNF-alpha response: by subtracting the respective backbones, it is clear that pCD-N1 was dominant in this kind of response. All data are shown as mean ± SD; * *p* < 0.05; ** *p* < 0.01 and *** *p* < 0.001 versus respective backbones.

Analysis of the cytokine responses in BALB/c mice serum samples indicated that the Ad5-N construct had more potential for IFN-gamma secretion. In addition, the BoHV4-∆TK-CCHFV-N and pCD-N1 groups demonstrated a relatively adequate amount of this cytokine (Figure 5A). When we analyzed the IL-2 responses, it was obvious that pCD-N1 is dominant. The other N-expressing constructs also elicited this response (Figure 5B). The results of IL-4, IL-5 and IL-13 were almost the same, while pCD-N1 showed more stimulation of the secretion of these cytokines in the serum samples. In addition, comparing the amount of these cytokines with the respective backbones clearly showed that the BoHV4-∆TK-CCHFV-N and Ad5-N groups possess a sufficient amount (Figure 5C–E). As an indicator of Th1/Th2 responses, we analyzed IL-6, IL-10 and TNF-alpha cytokines in the serum samples. BoHV4-∆TK-CCHFV-N was dominant in IL-6 and IL-10 secretion. In addition, pCD-N1 had the potential to sufficiently stimulate IL-10 response. On the other hand, this group demonstrated a relatively high amount of TNF-alpha in the immunized BALB/c mice on day 28 (Figure 5F–H).

**Figure 6:**
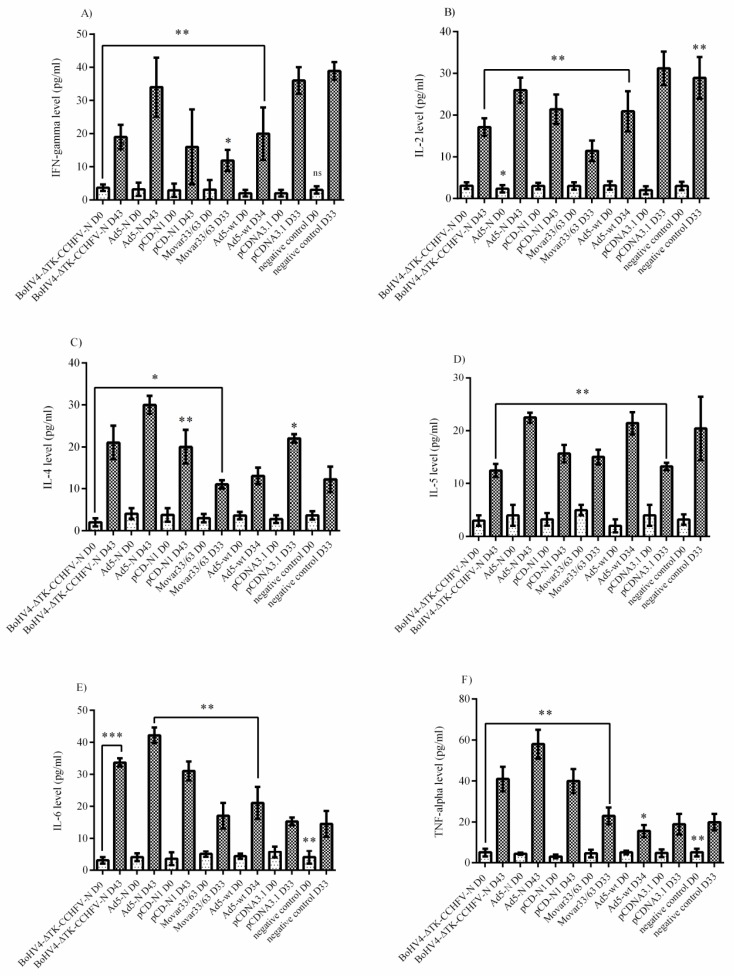
Cytokine responses in IFNα/β/γR−/− mice serum samples. (**A**) IFN-gamma response: no N-expressing constructs could stimulate adequate levels of this cytokine. (**B**) IL-2 response: the results were similar to IFN-gamma. (**C**) IL-4 response: the BoHV4-∆TK-CCHFV-N, Ad5-N and pCD-N1 groups’ responses were elevated. However, considering the respective backbone, only the BoHV4-∆TK-CCHFV-N and Ad5-N groups were significant. (**D**) IL-5 response: none of the three groups of BoHV4-∆TK-CCHFV-N, Ad5-N and pCD-N1 showed potential for IL-5 stimulation. (**E**) IL-6 response: the BoHV4-∆TK-CCHFV-N, Ad5-N and pCD-N1 groups demonstrated high levels. (**F**) TNF-alpha response: the results were similar to IL-6. All data are shown as mean ± SD; * *p* < 0.05; ** *p* < 0. versus respective backbones.

Analysis of the IFNα/β/γR−/− mice serum samples (performed on day 0 and the last survival days for N-expressing constructs or death days for control groups) showed that none of the N-expressing immunized groups could stimulate IFN-gamma, IL-2 and IL-5 secretion (Figure 6A,B,D). Despite the production of IL-4 in all three constructs (BoHV4-∆TK-CCHFV-N, Ad5-N and pCD-N1), the protective effect of this cytokine was only demonstrated in the BoHV4-∆TK-CCHFV-N and Ad5-N groups (Figure 6C). In addition, BoHV4-∆TK-CCHFV-N, Ad5-N and pCD-N1 stimulated relatively high levels of IL-6 and TNF-alpha induction, while negligible levels were observed in the control groups (Figure 6E,F).

### 4.5. Challenge Experiment, Antibody Passive Transfer and T Cell Adoptive Transfer in IFNα/β/γR−/−

**Figure 7:**
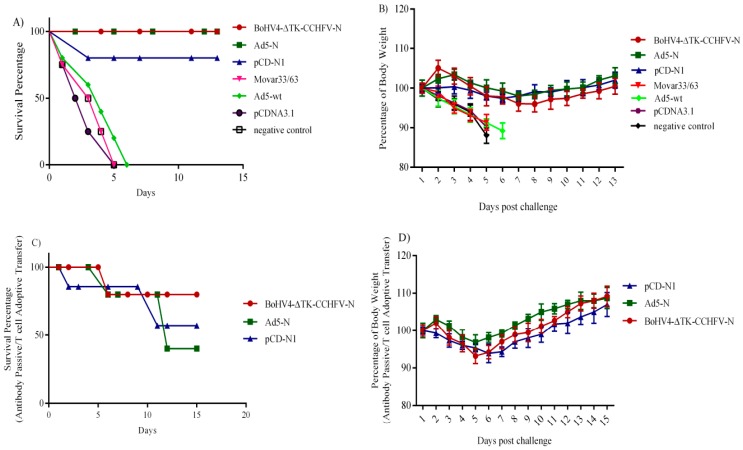
(**A**) Survival rate of immunized IFNα/β/γR−/− mice in challenge assay (1000TCID_50_/300 µL of Ank-2 strain): Survival rate of 100% observed in BoHV4-∆TK-CCHFV-N and Ad5-N groups. The results of pCD-N1 are also satisfactory with one death (survival rate of 75%). Control groups of Mover33/63 (death on day 5 post-challenge), Ad5-wt (death on day 6 post-challenge), pCDNA3.1 myc/His A (death on day 5 post-challenge) and normal saline (death on day 5 post-challenge) were also included in the experiment. (**B**) Percentage body weight of immunized IFNα/β/γR−/− mice after challenge experiment: despite lethal challenge, an almost stable body weight range was observed in the BoHV4-∆TK-CCHFV-N, Ad5-N and pCD-N1 groups. (**C**) Survival rates in the antibody passive (100–300 µL of serum containing 500 µg of IgG antibody) and T cell adoptive transfer (2 × 10^5^ splenocytes) experiment in IFNα/β/γR−/− mice: 24 h after transfer of splenocytes plus serum samples of BALB/c immunized mice, IFNα/β/γR−/− mice were challenged and BoHV4-∆TK-CCHFV-N showed a higher survival rate (75%) than Ad5-N and pCD-N1 (survival rate of 50%) after 15 days. (**D**) Percentage body weights of surviving IFNα/β/γR−/− mice in the antibody passive and T cell adoptive transfer experiment: As demonstrated, all surviving groups showed a decline in body weight percentage on day 4 post-challenge but stabilized after day 8 post-challenge. All data in Figure 7B,D are shown as mean ± SD.

After challenge with the Ank-2 strain, the BoHV4-∆TK-CCHFV-N and Ad5-N groups showed a 100% protection rate in IFNα/β/γR−/− mice previously immunized with two doses of vaccine constructs, while one mouse in the pCD-N1 group died due to virus replication confirmed by TCID_50_ assay from infected brain, spleen and liver collected on the death day. All control groups (Movar33/63, Ad5-wt, pCDNA3.1 myc/His A and normal saline) died within 6 days post-challenge (Figure 7A). In the surviving mice (vaccine groups), clinical symptoms including body weight loss (Figure 7B), ruffled fur, appearance changes, depression and nasal/ocular discharge, started on day 1 post-challenge and persisted for 3–4 days in different groups, followed by recovery in all survivors. The surviving mice’s conditions stabilized after 10 days post-challenge and the experiments were carried on for an additional 3 days to guarantee the observations. Virus replication in the control groups was also verified by TCID_50_ assay from collected organs (brain, spleen and liver) in SW-13 cells. Body weight percentages were recorded in all challenge groups as an indicator of clinical disease or recovery. In addition, to investigate the role of T cells and passive antibody transfer in protection against the lethal challenge, we conducted an antibody passive and T cell adoptive transfer assay in IFNα/β/γR−/− mice. In this experiment, 24 h after injection of the collected splenocytes plus serum samples of immunized BALB/c mice on day 28, IFNα/β/γR−/− mice received a lethal dose of CCHFV. Four groups of IFNα/β/γR−/−, each containing four individuals used in this assay, showed different protective rates. In the BoHV4-∆TK-CCHFV-N group, three mice survived (survival rate of 75%), while only two mice recovered (survival rate of 50%) from the challenge experiment in the Ad5-N and pCD-N1 groups (Figure 7C). The body weight percentages of the surviving animals from the antibody passive and T cell adoptive transfer experiment were documented as criteria for recovery. All groups showed recovery from illness based on body weight criteria on day 8 post-challenge. The results for BoHV4-∆TK-CCHFV-N and Ad5-N were clearly more satisfactory than pCD-N1 based on the stability of body weight changes and clinical symptoms (Figure 7D).

## 5. Discussion

In parallel with the incidence of tick-borne pathogens, there is evident expansion of CCHFV activity, with the virus being reported from several previously unaffected regions [24]. Due to the lack of a licensed vaccine or effective therapeutics, the development of new approaches to ensure protective immunity to CCHFV in susceptible individuals has become imperative. Previous efforts to develop immunity in indigenous populations of endemic regions, such as the Russian/Bulgarian vaccine construct, which contains chloroform-inactivated virus heated at 58 °C and absorbed on Al(OH)_3_, failed due to various drawbacks [25]. The main obstacle of the vaccine which was thoroughly evaluated for its efficacy in 2012 is the need for several booster doses to induce sufficient IFN-gamma response in volunteers [26]. In addition to employing inactivated viruses, various strategies have been explored for an effective CCHFV vaccine development such as targeting viral glycoproteins and the nucleocapsid using several DNA or virus-like particle-based vaccines [6,27,28].

In the present study, we set out to focus on the N protein for use in three different immunization platforms, namely, the adenoviral (Ad5-N) vector and a newly developed Bovine herpesvirus type 4 viral (BoHV4-∆TK-CCHFV-N) vector, alongside a DNA-based plasmid (pCD-N1). The CCHFV nucleocapsid protein (N), an essential component for intracellular virus replication, provides a prominent target for vaccine development as it is well conserved among global viral lineages and carries T cell epitopes. It was previously shown that the N-based vaccines of closely related viruses such as *Hantavirus* and Rift Valley fever virus had the potential to elicit sufficient immune responses for protection in challenge assays [29,30,31]. In addition, almost all vaccine development efforts based on the N protein of CCHFV have observed a protective rate of 100% in different expressing platforms and knock-out mice models [7,8,23,27].

The safety and flexibility of the viral vectors make them an interesting choice in both vaccination and oncolytic therapies. However, due to some obvious drawbacks of routinely used viral vectors, studies to develop new gene transfer systems are one of the most appealing research areas. For instance, a major obstacle of adenovirus-based vectors is the pre-existing immune response, which may cause inefficient expression following human vaccination. Employing rare virus serotypes, such as 2 and 5, as performed in this study, may overcome or alleviate this problem. In addition, adenovirus-based vectors are also highly immunogenic, so booster doses in individuals may induce serious side effects [32].

One of the crucial requirements of newly developed vaccine vectors is their capability to efficiently express genes of interest to specifically stimulate immune responses in the target animal model. It has previously been demonstrated that the BoHV-4-based viral vector can stimulate immune responses when expressing immunogenic dominant antigens of diverse viral diseases such as Ebola and Bovine herpesvirus type 1 [11]. For the first time, we have used this viral vector expressing an antigen from the *Bunyaviridae* family to examine its immunological potential against the CCHFV N protein in BALB/c and IFNα/β/γR−/− mice models. In addition, we developed two other constructs expressing the same antigen (Ad5-N and pCD-N1) to evaluate the superiority of the BoHV-4-based vaccine over them. Following the in vitro generation of the vaccine constructs, we immunized BALB/c and IFNα/β/γR−/−mice and the immune responses were analyzed via cytokine assays in virus-stimulated splenocytes (BALB/c) and serum samples (BALB/c and IFNα/β/γR−/−) and total/specific immunoglobulin subtypes in serum samples (BALB/c and IFNα/β/γR−/−). The neutralization potential of the immunoglobulins produced was further assessed via virus neutralization assays in both animal models. We observed that N-expressing systems (BoHV4-∆TK-CCHFV-N, Ad5-N and pCD-N1) could induce prominent cytokine responses in both BALB/c and IFNα/β/γR−/− mice after a two-dose injection of the vaccine constructs. In the BALB/c mice model, Ad5-N dominated in all cytokine responses over the other expression constructs in the supernatant of the virus-stimulated splenocytes. In addition, the BoHV4-∆TK-CCHFV-N construct further resulted in elevated IL-4, IL-6 and IL-10 responses in the same assay. The only significantly induced cytokine via pCD-N1 was IFN-gamma. On the other hand, the pCD-N1 construct showed high potential for IL-2, IL-4, IL-5, IL-13, IL-6 and TNF-alpha stimulation in the BALB/c mice serum samples. For a deeper investigation of the protective mechanism involved in the lethal challenge, we analyzed cytokine responses in IFNα/β/γR−/− mice serum samples on days 0, 28 (2 weeks after booster and before challenge) and different days post-challenge (death days in control mice and day 43 for the survivor groups). We observed a negligible potential of BoHV4-∆TK-CCHFV-N, Ad5-N and pCD-N1 to stimulate IFN-gamma, IL-2 and IL-5. In contrast, all three constructs showed high levels of IL-6 and TNF-alpha, which can be considered as associated with survival in the challenged mice.

To highlight the strength of the antibody responses in the recovered human patients, it is worth mentioning that IgM and IgG are serological markers of virus exposure that become detectable during or after the first week of the infection in the survivors. In fatal cases, a lack of IgG and IgM responses has been documented, indicating the impact of antibodies (although non-neutralizing) in the immune control of the virus replication [28,33,34]. In the serological assays of our study, the BoHV4-∆TK-CCHFV-N, Ad5-N and pCD-N1 constructs triggered the evaluated amount of total IgG immunoglobulin subtypes in BALB/c mice serum samples. We further evaluated the specific IgG antibody responses in IFNα/β/γR−/− mice (on day 28 and before the challenge experiment) by developing an in-house ELISA assay using CCHFV-infected cells and observed that pCD-N1 possessed more potential for humoral response stimulation. The other constructs also elicited significantly different IgG isotypes levels on day 28 (2 weeks after the second injection) that were analyzed by this assay. However, the total and specific antibody responses elicited by our N-expressing constructs failed to neutralize CCHFV in vitro, as assessed by the virus neutralization assay. The inability of the N protein to produce sufficient amounts of specific neutralizing antibodies was previously documented in knock-out animal models while non-neutralizing antibodies were also reported to protect mice from lethal CCHFV challenges [23,35,36].

In addition, in this study, we showed the role of T cell and passive antibody transfer of immunized BALB/c mice in CCHFV lethal challenge protection in IFNα/β/γR−/− mice models. As previously shown, balanced Th1 and Th2 responses are necessary to elicit protection in challenge experiments, so we decided to try a combination of T cell and antibody in this assay [8]. Antibody plus T cells of BoHV4-∆TK-CCHFV-N-immunized BALB/c mice elicited a 75% protection rate in IFNα/β/γR−/− mice during the challenge assay. This assay was previously conducted in the vaccinated mice by modified vaccinia Ankara expressing glycoprotein (MVA-GP), although no protection was documented [37].

Our findings indicate that, despite the variation between cytokine and antibody responses in BALB/c mice, immunization with the CCHFV-N-expressing BoHV-4 viral vector has a significant protection potential against lethal challenge in both immunized and T cells/antibody passive transfer IFNα/β/γR−/− mice. However, due to the similar protection rates observed in BoHV4-∆TK-CCHFV-N and Ad5-N, a detailed comparison of these platforms could not be performed. Further experimental evaluation of different aspects of the immune response, such as CD4 and CD8 T cells, is therefore needed. Our clinical findings following challenge further suggest that Ad5-N induced more immunity, which caused milder symptoms, leading to faster recovery in the IFNα/β/γR−/− mice. Due to some advantages of the BoHV-4-based viral vector over the DNA and viral vectors, this viral vector can be considered predominant in circumstances such as those in this study, in which all the constructs had the potential to sufficiently stimulate an immune response to protect against the lethal challenge [15]. One of the most important characteristics of BoHV-4 is its persistence in macrophages and monocyte cells. This may result in eliminating booster dose requirements due to frequent virus reactivation in persistence sites. In addition, macrophages and monocytes act as antigen-presenting cells, which can facilitate the presentation of antigens carried by the vector. However, the probable suppression of insert expression due to persistency resulting in insufficient immune stimulation must also be considered in the case of herpes viral vectors, including ours. The lack of a pre-existing immune response in humans and neutralizing antibodies in cattle, the main hosts of the virus, constitute additional advantages of BoHV-4-based delivery [15]. Therefore, BoHV-4 should be explored in detail as a new potential viral vector for CCHFV vaccination.

In conclusion, our findings indicate that the CCHFV N protein can be considered as the main target in vaccine development regardless of the expressing platform. We also established a new viral vector expressing viral N protein (BoHV4-∆TK-CCHFV-N), which showed considerable potential as an antigen delivery system. The next step will be to focus on the immunological aspects of this newly developed platform in mice, or even in other animal models such as goat or sheep, which are considered to play important roles in virus epidemiology in nature.

## Figures and Tables

**Table 1:** Primer sets used in PCR amplification reactions.

Name	Primer Sequence (5′→3′)	Purpose
CCHFV-N-F ^a^	Gaattcatggaaaacaagatcgagg	N-ORF amplification
CCHFV-N-R ^a^	Ctcgagaggaggagaaaagctgaa	
BoHV4-N-F ^b^	*Tggaagggtagagaggattgtctttgtgtccttctgtttgagagcaatgg*gggattttggtcatgaga	BoHV4-∆TK-CCHFV-N construction
BoHV4-N-R ^b^	*Tgctttgttgcagtttacaatacgtggagactcctgcaatattttacagc*gatttagagcttgacggg	
p516-SLiCE-N-F ^c^	**Tctccacaggtgtccactcccaggtccaaccgaattcccc**atggaaaacaagatcgagg	Ad5-N construction
p516-SLiCE-N-R ^c^	**Aaacaagttgctcgaagtcgacgagctcaagcttagatctccc**aggaggagaaaagctgaa	

^a^: Turkey-Kelkit06 S segment sequence (GenBank Accession Number: GQ337053). Underlined regular letters indicate restriction endonuclease recognition sites (5′:EcoRI and 3′:XhoI). ^b^: Underlined italic letters indicate the up- and downstream flanking sequences of the thymidine kinase gene (GenBank Accession Number: AF318573) of Bovine herpesvirus 4 (BoHV-4) to use in recombineering. Regular letters are homologous sequences to the pCDNA3.1 myc/His A vector to amplify the CMV-insert-polyA cassette. ^c^: Underlined bold letters indicate homologous arms to the pDC516 vector to use in the seamless ligation cloning extract (SLiCE) assay.

**Table 2:** Immunization schedule of BALB/c mice.

Group	Number	Inoculation Dose/Volume	Injection Route	Interval (Days)
BoHV4-∆TK-CCHFV-N	4	100TCID_50_/300 µL	i.p.*	0–14
Movar33/63	4	100TCID_50_/300 µL	i.p.	0–14
Ad5-N	4	100TCID_50_/300 µL	i.p.	0–14
Ad5-wt	4	100TCID_50_/300 µL	i.p.	0–14
pCD-N1	4	50 µg/100 µL	i.m.**	0–14
pCDNA3.1 myc/His A	4	50 µg/100 µL	i.m.	0–14
Normal saline	4	300 µL	i.p.	0–14

* intra-peritoneal, ** intra-muscular.
